# The novel prophage lysin Lys1459 exhibits broad-spectrum antibacterial activity via triple-binding domain

**DOI:** 10.1128/aem.01949-25

**Published:** 2026-06-11

**Authors:** Xiangpeng Yang, Yuan Wang, Xingshuai Li, Yixuan Li, Jingyi Wang, Xinyue Li, Xiaoting Yao, Xuguang Ruan, Juan Wang, Bin Ye, Jianrui Niu, Xinglin Zhang, Junfei Ma

**Affiliations:** 1 College of Agriculture and Forestry, Linyi University 165082, Linyi, China; University of Nebraska-Lincoln, Lincoln, Nebraska, USA

**Keywords:** lytic enzyme, *Streptococcus*, triple-binding activity, bacteremia model

## Abstract

Phage-encoded lysins represent a promising class of antimicrobial agents against drug-resistant bacteria due to their ability to degrade bacterial cell walls. This study characterizes a novel prophage-derived lysin, Lys1459, from *Streptococcus uberis*. We expressed and purified the full-length Lys1459 protein, along with its three individual domains (CHAP, A2, and CBD), to systematically evaluate its antibacterial spectrum, biochemical properties, and therapeutic efficacy. The full-length Lys1459 demonstrated potent lytic activity, achieving a 3-log reduction in *Streptococcus agalactiae* H-11-1 within 30 min at 50 μg/mL in Tris-HCl buffer. Notably, the three functional domains exhibited distinct binding spectra, which collectively contributed to a broad lytic range against streptococcal pathogens, including *S. agalactiae, S. dysgalactiae, S. uberis*, and *S. pyogenes*. In a mouse bacteremia model, intraperitoneal injection of Lys1459 significantly protected mice from lethal infection by *S. agalactiae* sgcDS001. Furthermore, the bacterial count of *S. agalactiae* in the blood of the treated mice was reduced by nearly three orders of magnitude. Our findings identify Lys1459 as a triple-binding-domain lysin with broad-spectrum bactericidal activity and robust *in vivo* efficacy, highlighting its potential as a therapeutic alternative against *S. agalactiae* infections.

**IMPORTANCE:**

A three-domain lysin, Lys1459, was identified in *Streptococcus uberis* SX-5-2. The differential binding specificities of the CHAP, A2, and CBD domains to bacterial cells likely underlie the enzyme’s broad-spectrum lytic activity against various *Streptococcus* species, including *S. agalactiae*, *S. dysgalactiae*, *S. uberis*, and *S. pyogenes.* A significant protective effect against lethal *S. agalactiae* infection was observed in mice treated intraperitoneally with Lys1459.

## INTRODUCTION

*Streptococcus agalactiae* is an opportunistic pathogen commonly colonizing the human gastrointestinal and genitourinary tracts, with an asymptomatic carriage rate of 9%–30% in healthy adults (1). *S. agalactiae* is capable of causing a variety of serious human infections and is a notable pathogen responsible for neonatal sepsis, pneumonia, and meningitis (2–4). It is worth noting that, in addition to posing a threat to human health, *S. agalactiae* is also the main causative agent of bovine mastitis (5). Bovine mastitis, one of the most common diseases in the global dairy industry, is primarily caused by microbial invasion of mammary tissue and results in significant economic losses to the livestock sector annually (6). Epidemiological investigations have shown that feeding contaminated milk to calves can lead to *S. agalactiae* colonization in the gastrointestinal tract or tonsils, a transmission route that contributes to persistent herd infections (7). Currently, antibiotics serve as the primary choice of therapy for both human infections and bovine mastitis caused by *S. agalactiae* (8). However, excessive antibiotic use has led to increasingly severe bacterial resistance (9). Previous studies have demonstrated that *S. agalactiae* strains isolated from human clinical samples and bovine mastitis cases in many regions have already developed resistance to antibiotics, such as erythromycin and tetracycline (10, 11). Therefore, the development of novel antimicrobial drugs to replace traditional antibiotics is not only crucial for achieving antibiotic-free dairy farming but also an urgent requirement to ensure the sustainable development of the livestock industry and safeguard human health.

Phage lysins represent a class of cell wall hydrolases expressed during the late phase of bacteriophage infection, exhibiting unique advantages, including stringent host specificity, potent antibacterial activity, low resistance induction rates, and synergistic effects with conventional antimicrobial agents (12). The outer membrane of Gram-negative bacteria typically poses a barrier that prevents endolysins from reaching the peptidoglycan layer. In contrast, the peptidoglycan in Gram-positive pathogens is readily accessible, making them susceptible to the exogenous application of endolysins. These characteristics position lysins as highly promising candidates for development into novel antimicrobial agents (13–15). Phage lysins can be derived from either lytic phages or prophages. Notably, due to the challenges in isolating lytic phages for *Streptococcus* species, prophage genomes have emerged as a crucial source for obtaining streptococcal lysins (16). Previous studies have identified several bacteriophage-derived lysins that exhibit antibacterial activity against *Streptococcus*. The lysin Lys1644, derived from *S. dysgalactiae* subsp. *dysgalactiae* lu24, exhibited species-specific activity, targeting *S. dysgalactiae* exclusively (17). In contrast, the lysin PlySK1249, derived from the *S. dysgalactiae* strain SK1249, exhibits efficacy against both *S. agalactiae* and *S. dysgalactiae*, as well as *S. pyogenes*, but demonstrates limited or no lytic activity against *S. uberis*. The therapeutic potential of PlySK1249 was confirmed in a murine model of *S. agalactiae* bacteremia, where a triple-dose regimen significantly improved survival rates (18). Collectively, these findings highlight the potential of naturally occurring lysins as novel therapeutics for bacterial infections, including bacteremia and other pathogenic conditions. In an era of rising antibiotic resistance, such lysins may serve as valuable alternatives.

In this study, a novel phage lysin, Lys1459, was identified from the *S. uberis* SX5-2 genome. The two lytic domains (CHAP, A2) and one binding domain (CBD) of this lysin both possess binding activity against different bacteria within the *Streptococcus* genus, which may explain the reason for its broad-spectrum lytic effect against *Streptococcus* species (including *S. agalactiae*, *S. dysgalactiae*, *S. uberis*, and *S. pyogenes*). Notably, Lys1459 exhibited significant therapeutic efficacy in a murine bacteremia model induced by *S. agalactiae*. These findings highlight Lys1459 as a promising antimicrobial candidate with cross-species lytic activity and clinical potential. This study provides critical theoretical support for developing phage lysin-based antimicrobials and offers novel strategic options for preventing and treating *S. agalactiae* infections.

## MATERIALS AND METHODS

### Bacteria and plasmids

All bacterial strains and plasmids used in this study are listed in Table S1. *Streptococcus* strains were cultured in brain heart infusion medium (BHI, Qingdao Haibo, Qingdao, China) at 37°C. *Escherichia coli* was cultured in Luria–Bertani medium (LB, Qingdao Haibo, Qingdao, China) at 37°C or 25°C.

### Genome sequencing and prophage analysis

The genomic DNA of *S. uberis* was extracted and purified using the TIANamp Bacteria DNA Kit (Tiangen, Beijing, China). The quantity and quality of the DNA were measured using a NanoDrop Spectrophotometer 2000 (Equl-Thermo Scientific, Waltham, MA, USA). The DNA samples were sent to Personalbio (Shanghai, China) and subjected to mechanical fragmentation for the construction of a whole-genome shotgun library. The library was subjected to sequencing using the Illumina MiSeq platform to generate paired-end (2 × 250 bp) reads through next-generation sequencing and the PacBio platform using third-generation single-molecule sequencing technology. Prophages in the genome were predicted by PHASTEST (19, 20).

### Construction of expression plasmids for Lys1459 and related proteins

The *lys1459* gene was PCR-amplified using primers pEC-lys1459-F/R and subsequently cloned into expression vectors pEC (with C-terminal 8× His tag) or pEG (containing both His and sfGFP tags) using a one-step cloning kit (Product No. C112-01, Vazyme Biotech Co., Ltd., Nanjing, China), generating recombinant plasmid pEC-lys1459 (17). The 3D structure of the lysin Lys1459 was predicted using the AlphaFold2 server (21). Using an identical strategy, we generated a series of plasmids encoding different domain combinations of Lys1459. The constructed plasmids included pEC-CHAP, pEC-A2-CBD, pEC-CHAP-A2, pEC-A2, pEG-CBD, pEG-A2, and pEG-CHAP. For detailed information about the pEC and pEG vectors and primer sequences, please refer to Fig. S1 and Table S1. All recombinant plasmids were transformed into *E. coli* DH5α or BL21 competent cells using a commercial transformation kit (Product No. B529303, Sangon Biotech Co., Ltd., Shanghai, China).

### Recombinant expression and purification of Lys1459 and its truncated proteins

The recombinant plasmids obtained as described above were transformed into *E. coli* BL21 (DE3) cells and cultured in LB medium supplemented with 50 μg/mL kanamycin at 37°C. When the OD_600_ reached a range of 0.4 to 0.6, 0.5 mM isopropyl-β-D-thiogalactopyranoside was added for induction, followed by incubation at 25°C for 16 h. Subsequently, the cells were harvested, washed, and lysed by sonication. The lysate was then purified using a Ni-IDA affinity chromatography column (Sangon Biotech, Shanghai, China). Finally, the purified protein Lys1459 and its related proteins (CHAP, A2-CBD, A2, CHAP-A2, CHAP-sfGFP, CBD-sfGFP, A2-sfGFP) were separated and analyzed by 12% sodium dodecyl sulfate-polyacrylamide gel electrophoresis (SDS-PAGE) to evaluate the purification efficiency and determine their molecular weights.

### Lytic activity of Lys1459 and its truncated proteins

To evaluate the lytic activity of Lys1459, the OD_600_ of *S. agalactiae* H-11-1 cells was adjusted to 0.8, and the cells were treated with different final concentrations (0, 25, 50, 75, and 100 μg/mL) of Lys1459 at 37°C. The OD_600_ was measured every 5 min for 1 h. All experiments were performed in triplicate. Similarly, we evaluated the lytic activity of Lys1459 (50 μg/mL) and its derived protein domains (CHAP, A2-CBD, A2, and CHAP-A2) against additional *Streptococcus* species.

### Optimal pH and temperature for Lys1459

To determine the optimal pH and temperature for Lys1459 activity, *S. agalactiae* cells were prepared in 50 mM Tris-HCl buffers at different pH values (ranging from 4 to 11) and temperatures (4°C, 25°C, 37°C, 42°C, and 56°C). The cell cultures were adjusted to an OD_600_ of 0.8. Subsequently, Lys1459 (final concentration: 50 μg/mL) was added to the cells, followed by incubation at 37°C. After 1 h, the OD_600_ was measured. Lytic activity (ΔOD_600_) was calculated as the difference between initial OD_600_ and OD_600_ after 1 h of treatment. All reactions were performed in triplicate.

### Lytic activity of Lys1459 in Tris-HCl buffer

To evaluate the lytic activity of Lys1459 in Tris-HCl buffer, equal amounts of *S. agalactiae* cells were prepared in a Tris-HCl buffer. Subsequently, Lys1459 was added at different final concentrations (0, 100, 200, and 400 μg/mL) and incubated at 37°C. After 1 h, the mixtures were serially diluted and plated on BHI solid agar. Following overnight incubation at 37°C, the colonies were counted. All experiments were performed in triplicate to ensure reproducibility and accuracy.

### Binding assessment of sfGFP fusion protein to the bacterial cell

To investigate the bacterial binding properties of different domains of Lys1459, we expressed and purified three fluorescent fusion proteins: CBD-sfGFP, CHAP-sfGFP, and A2-sfGFP. Furthermore, we evaluated the binding of these fusion proteins (CBD-sfGFP, CHAP-sfGFP, and A2-sfGFP) to various *Streptococcus* strains. Briefly, logarithmic-phase cells of each strain were harvested by centrifugation, resuspended in PBST buffer (50 mM NaH_2_PO_4_, 120 mM NaCl [pH 8.0], 0.01% Tween 20), and incubated with 10 to 20 ng of individual CBD-sfGFP proteins at room temperature for 5 min (22). Cells were then washed twice with PBST buffer, resuspended in fresh PBST, and subjected to confocal imaging analysis using a Nikon A1+ confocal laser scanning microscope (Nikon Instruments, Japan) equipped with a 60× oil-immersion objective (NA 1.40) (23).

### Protection from systemic *S. agalactiae* infection

Female BALB/c mice weighing 20 to 22 g (8 to 10 weeks of age) were purchased from the Jinan Pengyue Experimental Animal Breeding Co., Ltd. Groups of five mice per experiment were injected intraperitoneally (i.p.) with different inocula of *S. agalactiae* H-11-1 and *S. agalactiae* sgcDS001 (4 × 10^6^, 4 × 10^7^, 4 × 10^8^, and 4 × 10^9^ CFU/mouse) to determine the minimal dose that produced 100% mortality over a 3-day follow-up period (the minimal lethal dose [MLD]). The number of dead mice was recorded daily. Once the MLD had been determined, 1× MLD was used as the infective inoculum (challenge dose). The procedure for this experiment was carried out as described previously, with modifications (24, 25).

To determine the therapeutic efficacy of the lysin in the bacteremia model, a single dose of Lys1459 (200 μg) was administered via intraperitoneal injection 1 h post-bacterial challenge (24). Each dose group consisted of nine mice. For controls, an equivalent volume of saline was injected intraperitoneally 1 h after bacterial infection. An additional control group received i.p. Lys1459 (200 μg) 1 h following saline injection. To further evaluate the protective effect of Lys1459 against bacteremia, we performed bacterial colony counting. At designated time points, blood samples (10 μL) were collected from the tail veins of two groups of mice, the infection control group and the lysin-treated group, according to the following protocol: surviving mice were sampled at 48 h post-infection, while for mice that succumbed to infection, the bacterial count was defined as the CFU value from the final blood sample collected prior to death. The bacterial load was then quantified.

## RESULTS

### Identification of the lysin Lys1459 in the prophage genome of *S. uberis* SX5-2

**Fig 1:**
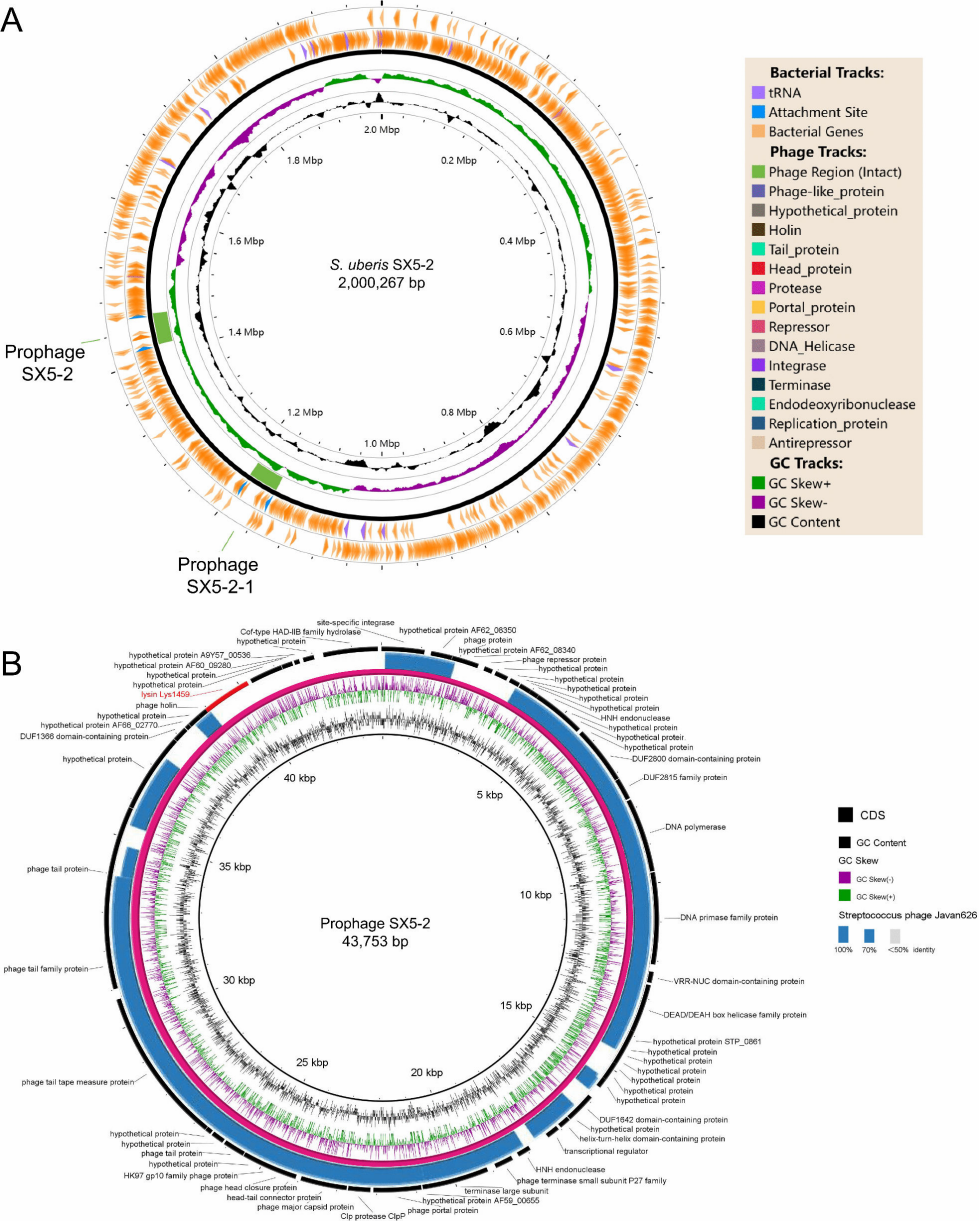
Genomic analysis of *S. uberis* SX5-2 and prophage SX5-2. (**A**) Whole-genome sequencing and prophage localization in *S. uberis* SX5-2. The prophage SX5-2 region is highlighted in green. (**B**) Circular genome map of prophage SX5-2. The red circle represents the genome of the putative phage SX5-2. The light blue circle (inner track) represents the homologous *Streptococcus* phage Javan626 genome; the height of the light blue bars indicates the sequence homology between phage SX5-2 and phage Javan626, with higher bars corresponding to greater homology. The outermost circle shows the annotated coding sequences (CDSs), among which the lysin gene Lys1459 is highlighted in red.

The complete genome of *S. uberis* SX5-2 is presented in Fig. 1A. The bacterial genome has a chromosomal span of 2,000,267 bp and encodes 1,998 predicted open reading frames (ORFs). Prophage prediction using PHASTEST indicated the presence of two phage sequences. One, a 42,047 bp prophage designated as SX5-2-1, was integrated between nucleotides 1,151,688 and 1,193,735. We attempted to express gene 1389, which is predicted to be a lysin protein located in this region, but this protein was not successfully expressed. The other, a 43,752 bp prophage designated as SX5-2, was integrated between positions 1,417,720 and 1,461,472 (Fig. 1A). Prophage SX5-2 exhibits a GC content of 39.25% and encodes 60 ORFs (Fig. 1B). Notably, annotation of prophage SX5-2 identified a lysin-encoding gene, which we designated Lys1459.

### Expression and purification of the modular lysin Lys1459

**Fig 2:**
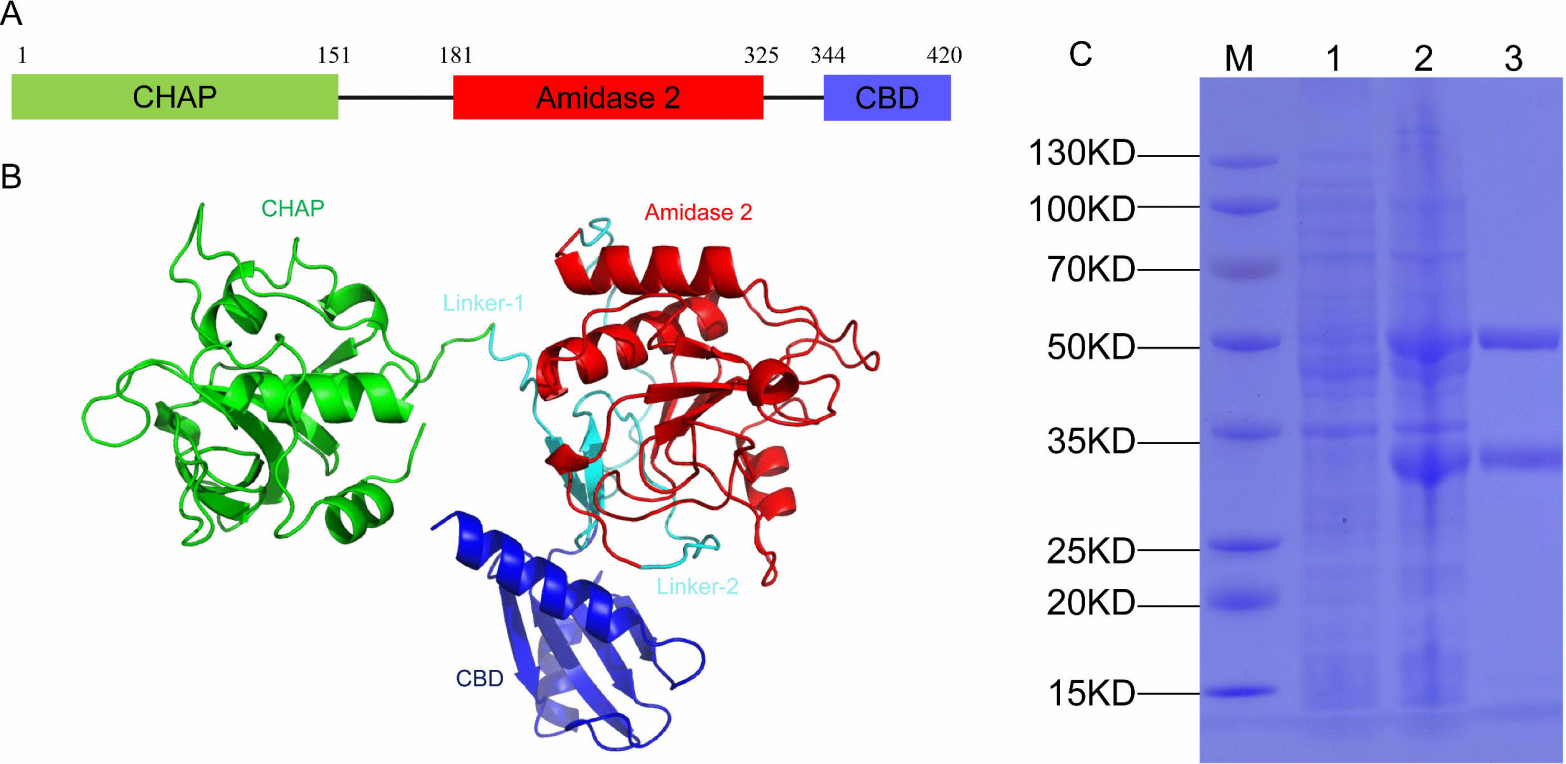
Structural analysis, protein expression, and purification of lysin Lys1459. (**A**) Domain architecture of Lys1459. (**B**) Predicted tertiary structure (AlphaFold2) of Lys1459 with color-coded functional domains: CHAP (green), Amidase-2 (red), and CBD (blue), connected by flexible linkers (light blue). (**C**) SDS-PAGE analysis of prokaryotic expression and purification of Lys1459: lane 1, total proteins from BL21(pEC) control; lane 2, total proteins from BL21/pEC-Lys1459; lane 3, purified Lys1459.

InterPro domain prediction (Fig. 2A) identified Lys1459 as a modular enzyme containing (i) an N-terminal CHAP lytic domain (1–151 aa) that cleaves amide bonds between glycan chains and peptides, (ii) a central Amidase-2 (A2) domain (181–325 aa) targeting the amide bond between N-acetylmuramic acid lactyl group and L-alanine α-amino group in cell wall degradation products, and (iii) a C-terminal cell wall-binding domain (CBD, 344–420 aa). AlphaFold2 structural prediction (Fig. 2B) revealed these domains form spatially independent units (CHAP [green], A2 [red], CBD [blue]) connected by flexible linkers (light blue), with high confidence scores (>90) for functional domains despite lower linker region confidence. SDS-PAGE analysis (Fig. 2C) demonstrated successful purification of Lys1459 from BL21(pEC-Lys1459) (lane 2), showing both the expected 50 kDa full-length protein and an additional ~32 kDa band that was absent in the BL21(pEC) control (lane 1). The extra band likely represents a fragment produced through secondary translation initiation.

### Lytic activity and stability of Lys1459

**Fig 3:**
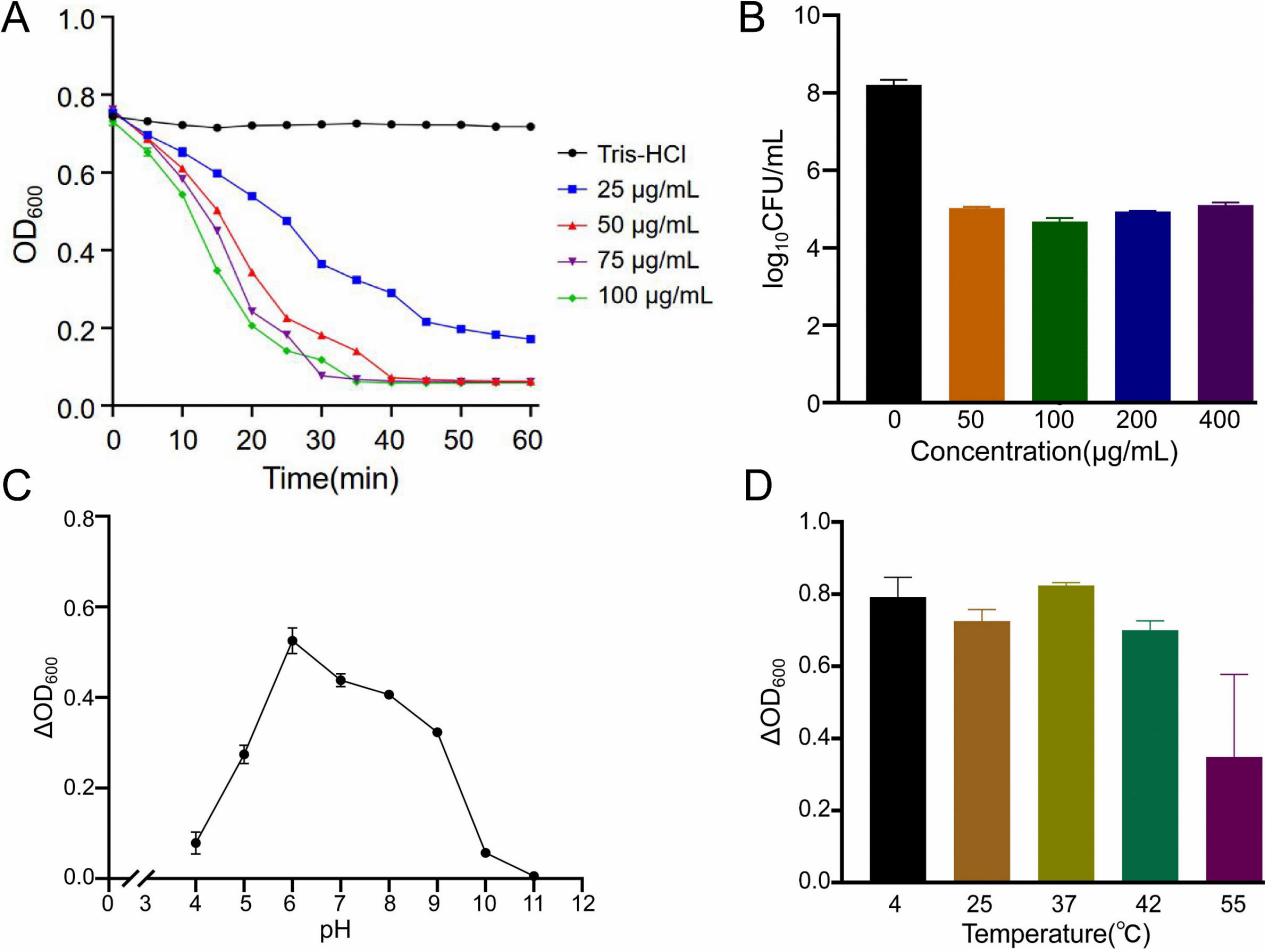
Lytic activity of Lys1459 against *S. agalactiae* H-11-1 and its stability under varying pH and temperature conditions. (**A**) Lysis curves of *S. agalactiae* H-11-1 treated with Lys1459 at different concentrations (0, 25, 50, 75, 100 μg/mL) in Tris-HCl buffer. (**B**) Bacterial reduction (by dilution plating) of *S. agalactiae* H-11-1 in Tris-HCl buffer after treatment with Lys1459 (0, 50, 100, 200, 400 μg/mL). (**C**) pH-dependent activity of Lys1459 in Tris-HCl (pH 4–11). Lytic activity (ΔOD_600_) was calculated as the difference between initial OD_600_ and OD_600_ after 1 h of treatment. Bars represent the standard error of the mean (SEM) of three experiments run in triplicate. (**D**) Temperature-dependent stability of Lys1459 in Tris-HCl (4°C–55°C), measured by ΔOD_600_.

The lytic activity of Lys1459 against *S. agalactiae* H-11-1 was determined by monitoring the OD_600_ reduction over time (Fig. 3A). Bacterial suspensions treated with varying concentrations of Lys1459 exhibited a gradual decline in OD_600_, whereas the control group (without lysin) showed no significant change. Lys1459 at 25 μg/mL exhibited a marginally weaker reduction trend. At concentrations ≥50 µg/mL, the OD_600_ decrease became consistent across groups, achieving substantial bactericidal effects within 40 min.

To evaluate the practical application of lysin Lys1459, we assessed its lytic activity against *S. agalactiae* in Tris-HCl buffer. As shown in Fig. 3B, treatment with 50 μg/mL Lys1459 reduced bacterial levels by approximately three orders of magnitude. Even at a higher concentration (400 μg/mL), Lys1459 achieved a similar reduction (~3 log units) in Tris-HCl buffe. These results indicate that Lys1459 exhibits strong bactericidal activity at relatively low concentrations, suggesting that Lys1459 has potential therapeutic value for combating *S. agalactiae* infections.

To determine the optimal pH and temperature for Lys1459 activity, we evaluated its lytic efficiency against *S. agalactiae* H-11-1 by monitoring OD_600_ reduction under varying conditions. Lys1459 exhibited minimal stability at pH 4 and 11, yet retained high stability over a broad range from pH 5 to 9, with a peak observed at pH 6 (Fig. 3C). The enzyme demonstrated substantial stability across temperatures of 4°C, 25°C, 37°C, and 45°C; however, a marked reduction in stability was noted at 55°C (Fig. 3D).

### Lytic activity of different domains of lysin Lys1459

**Fig 4:**
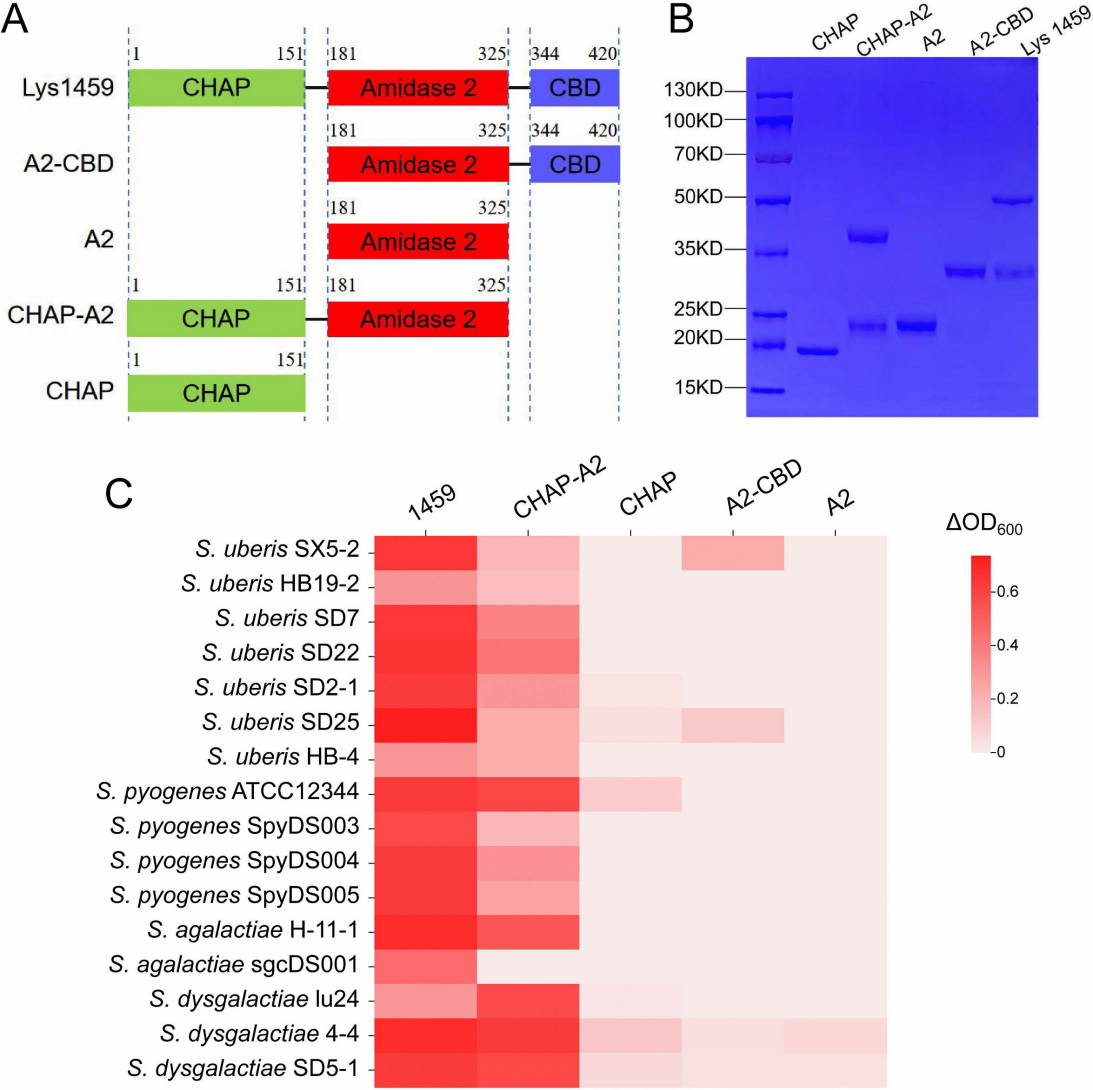
Domain architecture, SDS-PAGE analysis, and lytic activity of lysin Lys1459 and its truncated variants. (**A**) Schematic representation of protein constructs. Functional domains are color-coded: CHAP (green), A2 (red), and CBD (blue), with amino acid positions numbered. (**B**) SDS-PAGE analysis of purified proteins. (**C**) Lytic activity against *S. agalactiae* H-11-1 (50 μg/mL), measured as ΔOD_600_ (initial 1 h post-treatment).

To determine the lytic effects of distinct domains and their combinations in Lys1459 on different bacterial strains, we constructed and expressed five protein variants (Lys1459, CHAP, CHAP-A2, A2, and A2-CBD), followed by purification of each protein construct. The schematic domain structures are illustrated in Fig. 4A. SDS-PAGE analysis revealed that all proteins achieved >90% purity, except for the two CHAP-containing constructs (Lys1459, CHAP-A2), which consistently exhibited co-purified shadow bands at high concentrations, as shown in Fig. 4B. The molecular weights of these faint bands correspond to fragments terminating at identical residues in different constructs (e.g., a secondary band of Lys1459 comigrates with the band of A2-CBD, while the band of CHAP-A2 comigrates with the band of A2). This suggests the potential existence of preferential proteolytic cleavage sites or secondary translation initiation sites. It has been reported that some lysins possess internal translation initiation sites that can produce short variant isoforms, as well as full-length lysins (26). Our analysis identified a putative internal translation initiation site in the CHAP domain, located 36 bp upstream of its stop codon (Fig. S2).

We subsequently evaluated the lytic activity of these proteins against *S. agalactiae*, *S. uberis*, *S. pyogenes*, and *S. dysgalactiae* (Fig. 4C). The results demonstrated that the individual domains (CHAP, A2) and the A2-CBD combination showed only marginal activity against select strains. In contrast, the CHAP-A2 construct exhibited strong lytic activity. Although the full-length Lys1459 exhibited significant lytic activity against all tested bacterial strains, it is noteworthy that CHAP-A2 demonstrated superior activity compared to the full-length enzyme against *S. dysgalactiae* lu24, despite minimal activity observed for either the CHAP or A2 domain alone against this strain.

### Specific binding of CHAP, A2, and CBD domains to various *Streptococcus* strains

**Fig 5:**
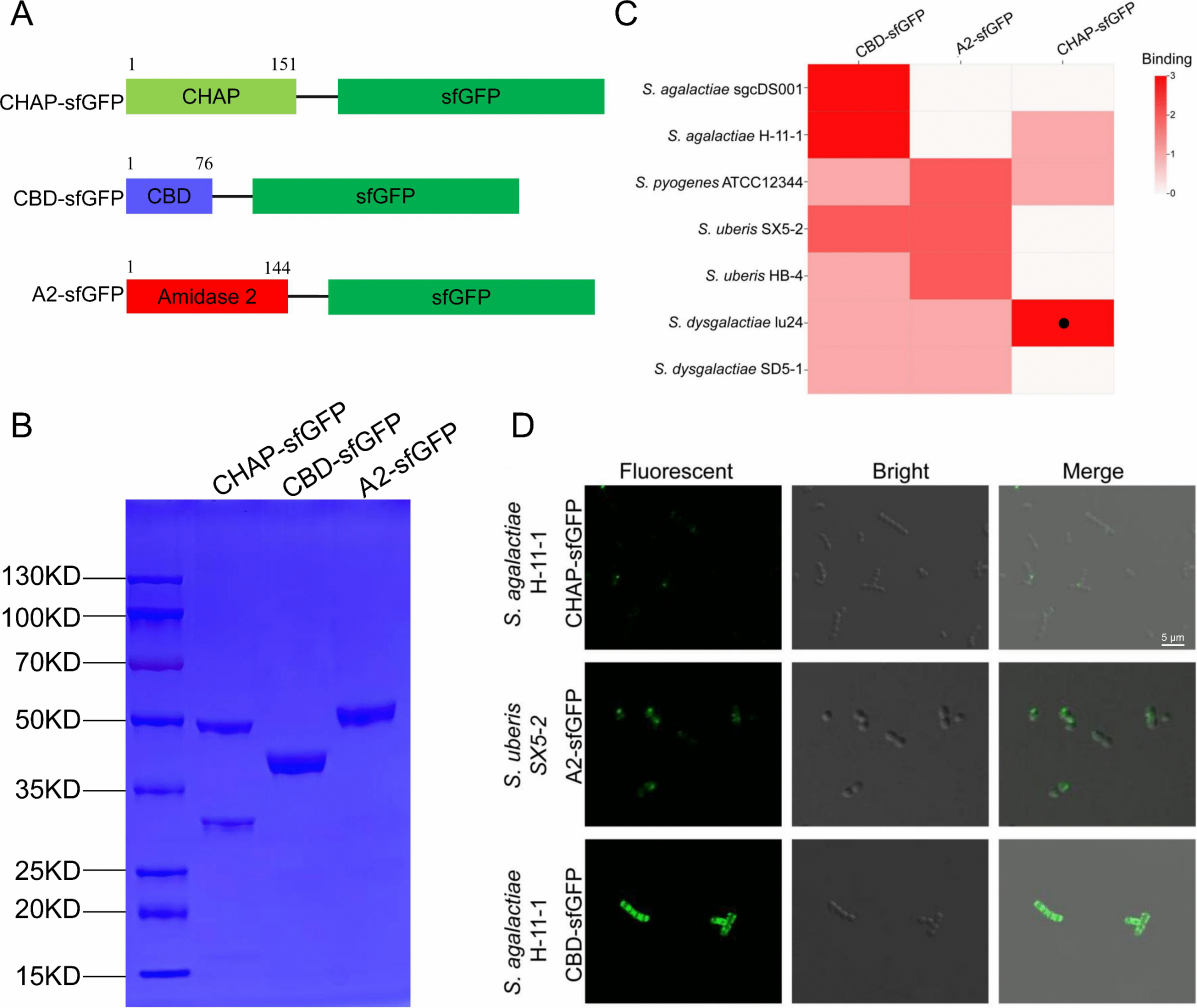
Fusion expression and binding activity assay of Lys1459 domains with sfGFP. (**A**) Schematic representation of protein constructs. Functional domains are color-coded: CHAP (light green), A2 (red), CBD (blue), and sfGFP (green), with numbering indicating amino acid positions. (**B**) SDS-PAGE analysis of purified proteins. (**C**) Confocal microscopy images demonstrating the binding activity of the three fusion proteins to bacterial cells. The binding activity was categorized into three levels based on fluorescence intensity (brightness of fluorescent protein bound to bacterial strains) and binding rate (proportion of bacteria bound to the fluorescent protein relative to the total bacterial population). Weak binding (level 1): low fluorescence intensity and/or low binding rate. Moderate binding (level 2): intermediate fluorescence intensity and binding rate. Strong binding (level 3): high fluorescence intensity and high binding rate. The circular black zones indicate that the fusion protein caused lysis of the bacterial strain. Scale bar represents 5 μm (the same scale bar applies to all images). (**D**) The representative images demonstrate that different structural domains of Lys1459 all possess the ability to bind bacteria.

To determine the binding activities of the CHAP, A2, and CBD domains of Lys1459 protein to different bacteria, the predicted CHAP, A2, and CBD domains were separately cloned into the pEG vector and purified. The schematic diagrams of the CHAP-sfGFP, A2-sfGFP, and CBD-sfGFP constructs, as well as the SDS-PAGE analysis showing the purification of these fusion proteins, are presented in Fig. 5A and B, respectively. Purified proteins (CHAP-sfGFP, A2-sfGFP, and CBD-sfGFP) were added to treated bacterial cells, and binding was visualized using confocal microscopy. CBD-sfGFP showed strong binding activity to *S. agalactiae* H-11-1, *S. agalactiae* sgcDS001, and *S. uberis* SX5-2, but only weak activity against *S. uberis* SX5-2, *S. uberis* HB-4, *S. pyogenes* ATCC12344, *S. dysgalactiae* lu24, and *S. dysgalactiae* SD5-1. Next, we tested A2-sfGFP’s binding activity to these strains. The results revealed strong binding to *S. uberis* SX5-2, *S. uberis* HB-4, and *S. pyogenes* ATCC12344, but weak binding to *S. dysgalactiae* lu24 and *S. dysgalactiae* SD5-1. No binding activity was observed for *S. agalactiae* H-11-1 or *S. agalactiae* sgcDS001. CHAP-sfGFP exhibited weak binding only to *S. agalactiae* H-11-1 and *S. pyogenes* ATCC12344 (Fig. 5D). Unexpectedly, CHAP-sfGFP completely lysed *S. dysgalactiae* lu24 (Fig. 5C and Fig. S3 ). While InterPro predicts that the A2 domain primarily functions to cleave the amide bond between N-acetylmuramoyl and L-amino acids in bacterial cell walls, intriguingly, our results demonstrate its previously unrecognized binding specificity toward certain bacterial species.

### Lys1459 efficacy in a mouse model of *S. agalactiae*-induced bacteremia

The results of intraperitoneal injection of two *S*. *agalactiae* strains in mice showed that, even with a dose of 4 × 10^9^ CFU of *S. agalactiae* strain H-11-1, no complete mortality of mice was observed. However, the sgcDS001 strain exhibited significantly higher virulence, as doses of both 4 × 10^8^ and 4 × 10^9^ CFU per mouse induced sustained bacteremia and resulted in 100% mortality within 2 days, as shown in Fig. S4. Therefore, we selected *S. agalactiae* sgcDS001 for the mouse bacteremia model experiments, and the chosen dose was approximately 4 × 10^8^ cells per mouse, which is the average MLD.

**Fig 6:**
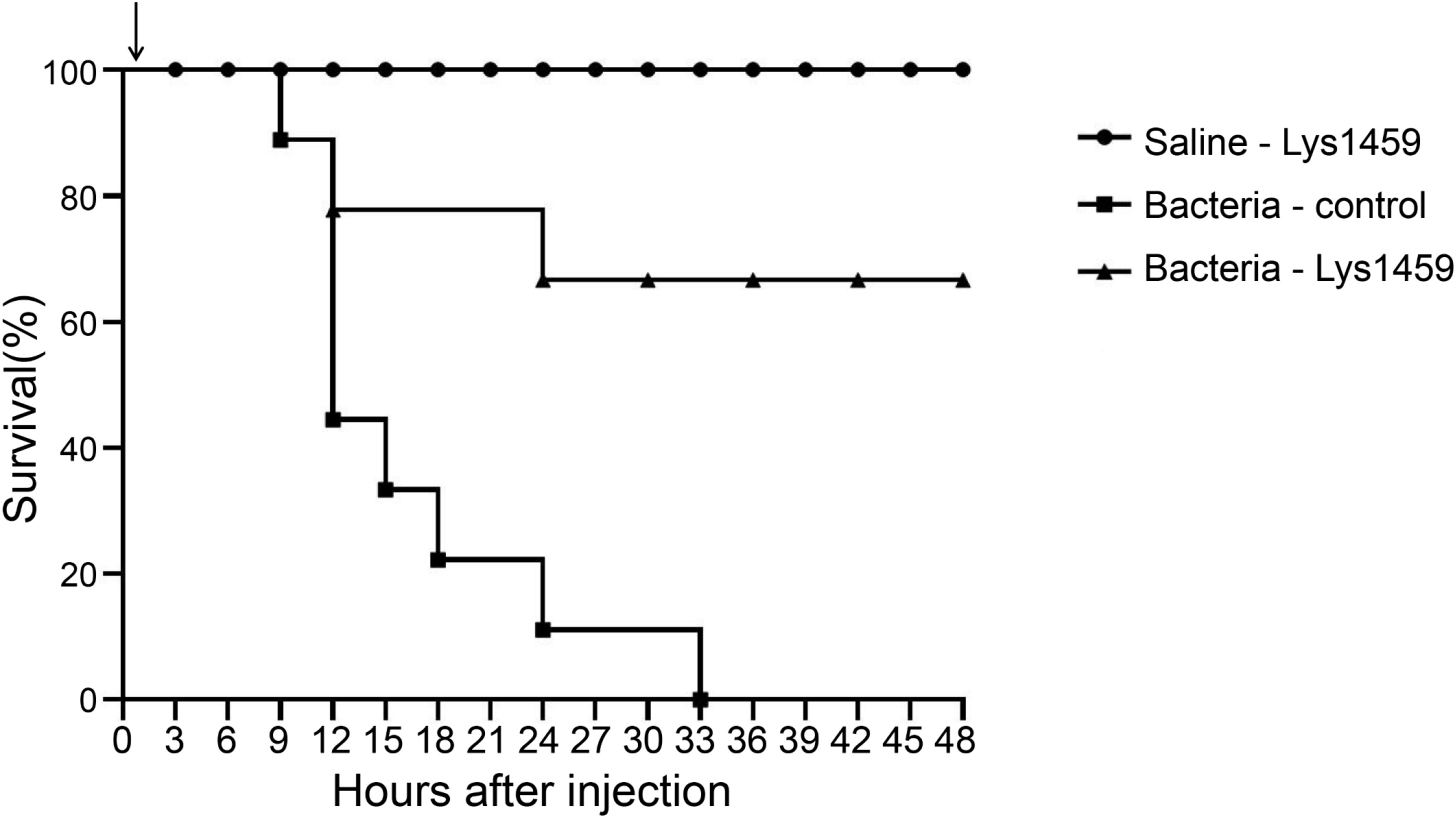
Lys1459 protects mice from lethal *S. agalactiae* sgcDS001 infection. Bacteria-Lys1459: mice were i.p. injected with 1× MLD of *S. agalactiae* sgcDS001, followed by i.p. administration of 200 μg Lys1459 at 1 h post-infection (black triangles). Bacteria-control: mice received i.p. injection of 1× MLD *S*. *agalactiae* sgcDS001, followed by i.p. saline (control) at 1 h (black squares). Saline-control: mice were injected i.p. with saline instead of bacteria, followed by 200 μg Lys1459 at 1 h (black circles).

As shown in Fig. 6, all nine mice in the control group (injected with bacteria alone) died within 33 h. Intraperitoneal administration of 200 μg Lys1459 at 1 h post-infection provided complete protection in two-thirds of the treated mice (survival rate: 67%), demonstrating its life-saving efficacy. Furthermore, no mortality was observed in the saline control group receiving 200 μg Lys1459, and no adverse effects were detected at this single dose. Bacterial load in the blood was assessed by CFU counts. As shown in Fig. S5, lysin treatment resulted in a rapid and significant reduction in bacterial load compared to the control group. These data directly demonstrate the efficacy of the lysin in controlling infection *in vivo*, which strongly correlates with the observed survival benefit.

## DISCUSSION

The development and spread of antimicrobial resistance not only pose a significant threat to human health (27) but also present substantial challenges for veterinary health management (28). Consequently, developing novel antimicrobial agents has become an urgent priority. Lysins derived from phages are typically identified by screening and sequencing phages that target specific bacteria, and a significant proportion of these lysins originate from temperate phages (29, 30). This provides an effective approach for obtaining streptococcal lysins. Through heterologous expression of these lysin genes, their lytic activity against target bacteria can be systematically evaluated. Previous studies have shown that several lysins, including Lys1644, PlyC, Ply700 (17, 31, 32), PlySK1249 (18), as well as PlySs2 and PlySs9 (33), λSA2, and B30 (34), all fail to lyse *S. uberis*, *S. dysgalactiae*, *S. agalactiae*, and *S. pyogenes* individually. In striking contrast to these findings, the single enzyme Lys1459 demonstrated significant bactericidal activity against all tested pathogens, including *S. uberis*, *S. dysgalactiae*, *S. agalactiae*, and *S. pyogenes*. Therefore, as a single lysin with broad-spectrum lytic activity, Lys1459 shows great potential as a novel antimicrobial agent against streptococcal infections.

The broad-spectrum lytic mechanism of lysin Lys1459 was found to depend on interdomain synergy, not the action of a single domain. Initially, we characterized the CBD domain and observed specific binding to *S. agalactiae* and *S. uberis*, aligning with the reported specificity of the Ply700 CBD. However, its affinity for *S. pyogenes* and *S. agalactiae* was relatively weak, suggesting that other domains of Lys1459 may possess binding functionality. To probe this further, we expressed the A2 and CHAP domains as fluorescent fusion proteins. The results revealed that while the A2 domain bound *S. uberis*, *S. pyogenes*, and *S. dysgalactiae*, its binding to *S. agalactiae* was weak. Most notably, the isolated CHAP domain displayed two key features: first, mild binding activity against certain strains of *S. agalactiae* and *S. pyogenes*; and second, the ability, as a CHAP-sfGFP fusion, to mediate complete lysis of *S. dysgalactiae* lu24 independently of the A2 and CBD domains. Consequently, we hypothesize that CHAP has a dual function, encompassing both lytic and specific binding activities. In conclusion, the combined binding capabilities of the CBD, A2, and CHAP domains to different streptococci strongly support a model where the broad-spectrum efficacy of Lys1459 is conferred by its multi-domain architecture, with each domain targeting distinct bacteria to collectively broaden the antimicrobial range.

Our structural analysis confirmed that Lys1459 has dual lytic domains (CHAP and A2) and a CBD, similar to endolysins, such as PlyTW and LysK (35, 36). Functional characterization of the truncated derivatives revealed the independent and synergistic roles of these domains. As expected, the isolated A2 domain was inactive, which is consistent with previous findings for PlyTW. However, interestingly, the A2-CBD truncation retained weak but detectable lytic activity against *S. uberis* strains, contrasting with the completely inactive A2-SH3b unit in LysK. Moreover, the truncated CHAP domain alone was sufficient to lyse a variety of *Streptococcus* strains, a finding consistent with the typical function of CHAP domains in related endolysins (35–37). Notably, in experiments targeting *S. dysgalactiae* lu24, the CHAP-A2 dual domain performed significantly better than the full-length protein. We speculate that, in this specific case, the CBD in the full-length enzyme may create steric hindrance, thereby impeding the lytic efficiency or substrate accessibility of the CHAP or A2 domains. In summary, our data support a model of functional synergy. The CHAP domain serves as the primary lytic domain, but its optimal activity is enhanced by the A2 domain. Although the CBD is dispensable for activity against certain strains and even counterproductive against *S. dysgalactiae* lu24, it might be crucial for binding to other bacterial targets. This domain synergy underscores the evolutionarily tailored regulation of endolysin function for specific host bacteria.

Lys1459 exhibits a relatively broad optimal pH range of 5.5–7.0, which is different from the pH ranges of previously reported lysins, such as B30 (4.5–6) (37), PlyGBS (4–6) (38), PlySK1249 (7.0–8.5) (18), and PlyCYU (7.0–9) (39). This characteristic enables it to function effectively across diverse infectious microenvironments. Moreover, the enzyme demonstrated potent bactericidal activity in Tris-HCl buffer, reducing the viable count of *S. agalactiae* H-11-1 by 3 log units (99.9%) within 1 h (Fig. 3B). Additionally, Lys1459 maintained stability over a wide temperature range from 4°C to 42°C, covering conditions from mammalian physiological temperatures to refrigerated food storage. The broad stability of this lysin under varying pH and temperature conditions collectively underscores its potential for both *in vivo* therapeutic applications and use in the dairy industry.

Intraperitoneal injection of Lys1459 significantly protected mice from lethal infection with *S. agalactiae* sgcDS001. Meanwhile, results from tail vein blood collection showed that the bacterial count in the treated mice was reduced by nearly three orders of magnitude. This finding strongly suggests that the potent antimicrobial efficacy of Lys1459 underlies its protection of mouse survival, as it effectively controls the systemic infection by directly reducing the pathogenic bacterial count. These findings strongly support that Lys1459, as a novel *S. agalactiae*-specific endolysin, possesses significant clinical therapeutic potential, providing critical experimental evidence for the development of new treatment strategies against *S. agalactiae* infections.

### Conclusions

This study reports the discovery and characterization of a novel phage lysin, Lys1459, with potent antimicrobial properties. Our findings demonstrate that fusion of its three functional domains (CHAP, A2, and CBD) confers specific binding activity against various bacterial strains, likely explaining its broad-spectrum lytic activity against *Streptococcus,* including *S. agalactia*e, *S. dysgalactiae*, *S. uberis*, and *S. pyogenes*. In a mouse bacteremia model, intraperitoneal injection of Lys1459 significantly protected mice from lethal infection by *S. agalactia*e sgcDS001. Furthermore, the bacterial count of *S. agalactiae* in the blood of the treated mice was reduced by nearly three orders of magnitude. These pivotal findings provide compelling evidence for developing innovative antimicrobial strategies, positioning Lys1459 as a promising phage-derived lysin therapeutic that could serve as an alternative to conventional antibiotics for *S. agalactiae* infections.

## Data Availability

The whole genome sequences have been submitted to the NCBI under the accession number PRJNA1262693. The authors confirm that all data underlying the findings are fully available without restriction.

## SUPPLEMENTAL MATERIAL

Fig. S1 aem.01949-25-s0001.tif Lysin expression plasmids pEC(A) and pEG(B).

Fig. S2 aem.01949-25-s0002.tif Gene map of the CHAP-A2-CBD fusion construct.

Fig. S3 aem.01949-25-s0003.tif Binding analysis of CHAP, A2, and CBD fused to sfGFP to bacterial cells by confocal microscopy.

Fig. S4 aem.01949-25-s0004.tif Mouse survival in the *S. agalactiae* infection model.

Fig. S5 aem.01949-25-s0005.tif Bacterial loads in blood in a murine model of *S. agalactiae* bacteremia established by intraperitoneal challenge.

Supplemental legends aem.01949-25-s0006.doc Descriptive legends for Fig. S1 to S5.

Table S1 aem.01949-25-s0007.xls Strains used in this study.

ASM does not own the copyrights to Supplemental Material that may be linked to, or accessed through, an article. The authors have granted ASM a non-exclusive, world-wide license to publish the Supplemental Material files. Please contact the corresponding author directly for reuse.
