# Supplementary material for: The novel prophage lysin Lys1459 exhibits broad-spectrum antibacterial activity via triple-binding domain
Source: Appl Environ Microbiol. 2026 Jun 11;92(7):e01949-25. doi: 10.1128/aem.01949-25 (PMC13390383; doi:10.1128/aem.01949-25)
Supplement: Supplemental legends — Descriptive legends for Fig. S1 to S5. [file aem.01949-25-s0006.doc]

Fig.S1 The lysin expression plasmids pEC(A) and pEG(B).

(A) Map of the pEC vector (5302 bp), containing the lac operon, a kanamycin resistance marker (KanR), an origin of replication (ori), and an 8×His tag. (B) Map of the pEG vector (6012 bp), derived from pEC, which contains the sfGFP coding sequence in addition to the core regulatory and replication elements present in pEC.





Fig.S2 Gene map of the CHAP-A2-CBD fusion construct.

The construct contains the CHAP (green), A2 (red), and CBD (blue) domains within a single open reading frame, along with the start codon (ATG) and a predicted ribosome binding site (RBS).





Fig.S3 Binding analysis of CHAP, A2, and CBD fused to sfGFP to bacterial cells by confocal microscopy.

Binding of CHAP-sfGFP, A2-sfGFP, and CBD-sfGFP fusion proteins to *S. agalactiae* (H-11-1, sgcDS001), *S. uberis* (SX5-2, HB-4), *S. dysgalactiae* (lu24, SD5-1), and *S. pyogenes* ATCC12344 was analyzed by confocal microscopy. Three images are shown for each strain: fluorescence (sfGFP signal), bright-field, and merged images. The red box highlights the lytic effect of CHAP-sfGFP on *S. dysgalactiae* lu24, observed as disruption of cellular morphology. Scale bar represents 5 μm. (The same scale bar applies to all images.)





Fig.S4 Mouse survival in the *S. agalactiae* infection model.

Survival curves of mice following injection with 4×10⁶, 4×10⁷, 4×10⁸, or 4×10⁹ CFU of (A) S. agalactiae H-11-1 or (B) S. agalactiae sgcDS001. S. agalactiae sgcDS001 exhibited higher virulence, causing 100% mortality within 48 hours at a dose of 4×10⁸ CFU, whereas S. agalactiae H-11-1 resulted in only partial mortality at the highest dose (4×10⁹ CFU) over the 72-hour observation period.





Fig.S5 Bacterial loads in blood in a murine model of *S. agalactiae* bacteremia established by intraperitoneal challenge.

Mice were intraperitoneally challenged with approximately 4×10^8^ CFU of *S. agalactiae* strain sgcDS001. Blood samples were collected via the tail vein at the indicated time points, serially diluted, and plated on BHI agar for colony counting. Each symbol represents the bacterial load (CFU/mL) from an individual mouse. Filled circles (●) denote data points from mice that succumbed to infection, representing the last sampling time point before death. Black squares (■) denote data points from surviving mice, collected at the experimental endpoint (48 hours post-infection). The horizontal bars indicate the median for each group.
